# Supplementary material for: Maintenance of Flap Endonucleases for Long-Patch Base Excision DNA Repair in Mouse Muscle and Neuronal Cells Differentiated In Vitro
Source: Int J Mol Sci. 2023 Aug 12;24(16):12715. doi: 10.3390/ijms241612715 (PMC10454756; doi:10.3390/ijms241612715)
Supplement: Supplementary file 1 [file ijms-24-12715-s001.zip › ijms-2430461-supplementary.pdf]

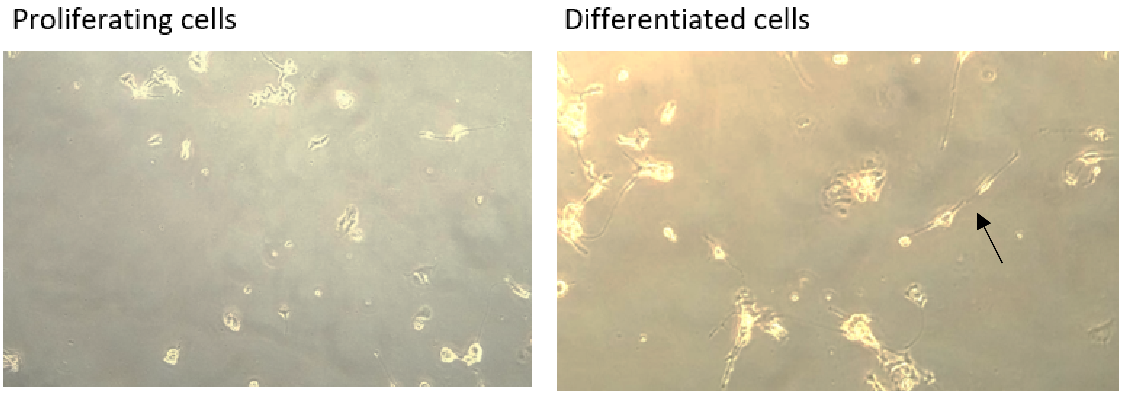

Figure S1. Micrograph of CAD cells. A single dish of prolifetating CAD cells (left) was differentiated by ommision of Fetal Bovine Serum from medium for 5 days (right). Arrow indicates an example of dentrite formation.

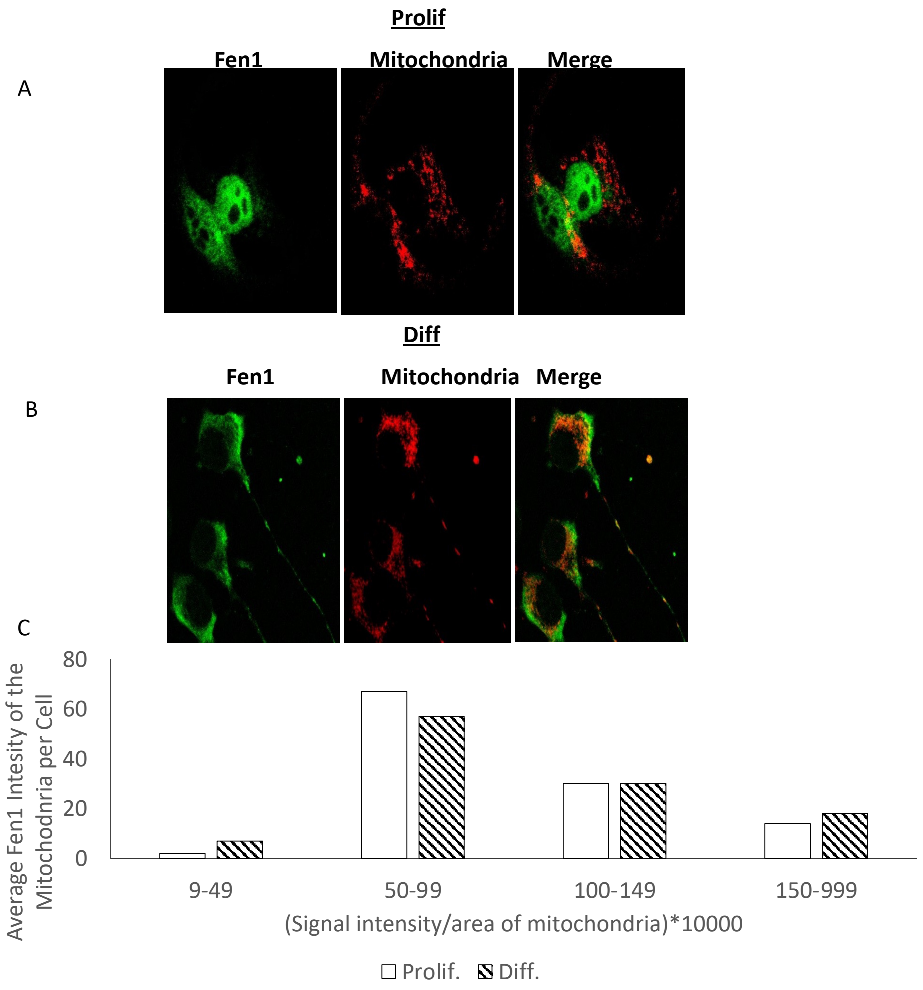

Figure S2. Immunofluorescent detection of Fen1 in CAD cells. Cells were incubated with Mitotracker Red, then fixed with 4% paraformaldehyde and labeled with a primary antibody against Fen1 and a secondary antibody tagged with FITC. Representative examples are shown of flattened Z-stack images: **A**, Proliferating cells (Prolif); and **B**, Differentiated (Diff). **C**, Quantification using Image J. Quantification was completed by measuring the intensity of Fen1 within the mitochondria, excluding any signal that did not co-localize with Mitotracker Red. Mitochondrial Fen1 was quantified for each cell, then averaged for the number of cells. The signal intensities were then binned into 4 portions.

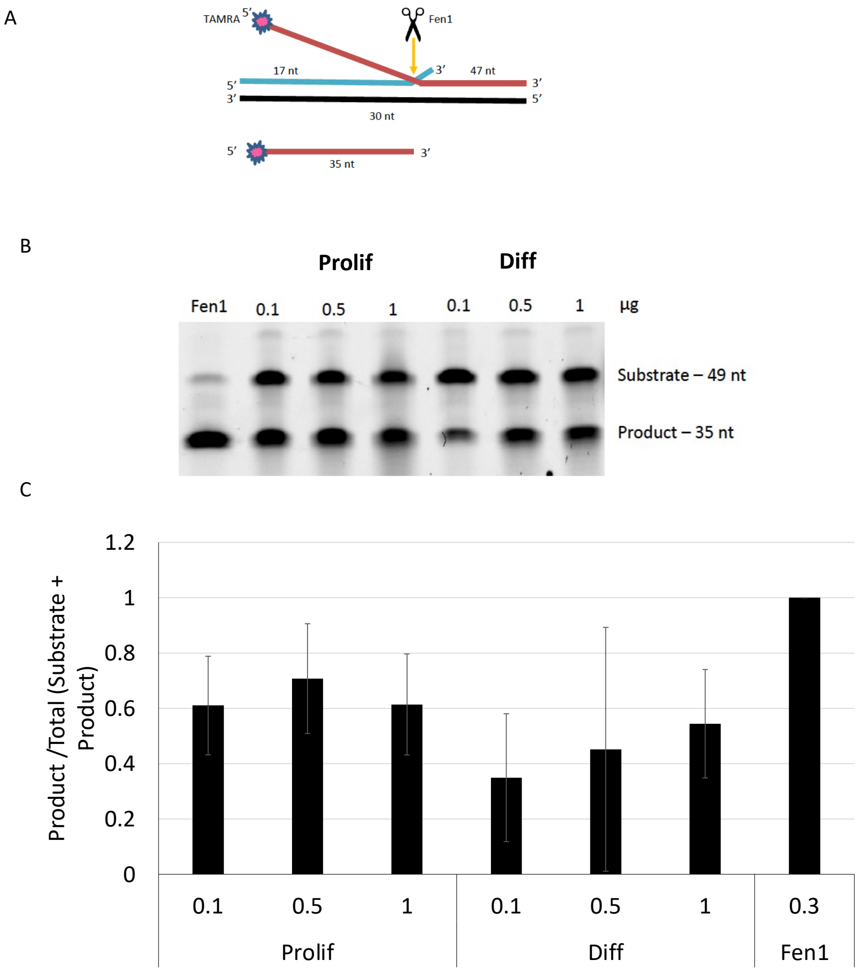

Figure S3. Flap excision activity in CAD whole cell extracts using a 5′-labeled substrate. A, in this structure, the target flap strand was labeled on its 3′ end with the TAMRA fluor. The expected Fen1 product is shown. B. Representative gel for the flap excision assay of whole-cell extracts from proliferating (Prolif.) and differentiated (Diff.) CAD cells. Increasing concentrations of extract were used with 1 pmol of substrate. C. Quantification of 3 independent experiments using Image J program.

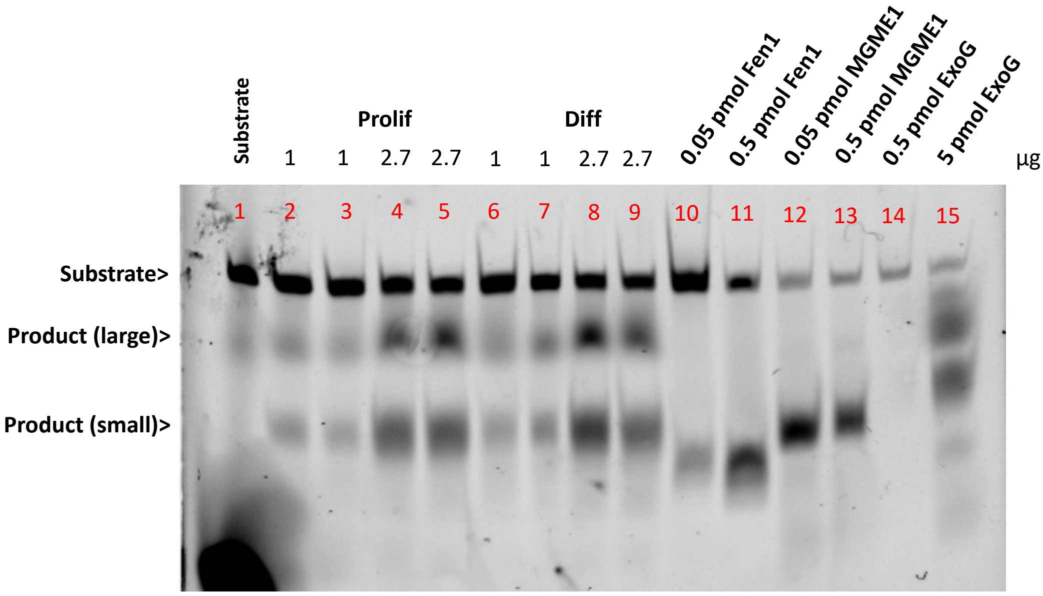

Figure S4. Original gel of the flap excision activity using mitochondrial extracts from Prolif. and Diff. CAD cells. Specific conditions were run in duplicates. Lanes 4, 8, 11, 13, and 15 are represented in Fig. 4. The flap substrate (1 pmol) was incubated with the indicated amount of mitochondrial extract, Fen1, MGME1, or ExoG at 37°C for 15 min.

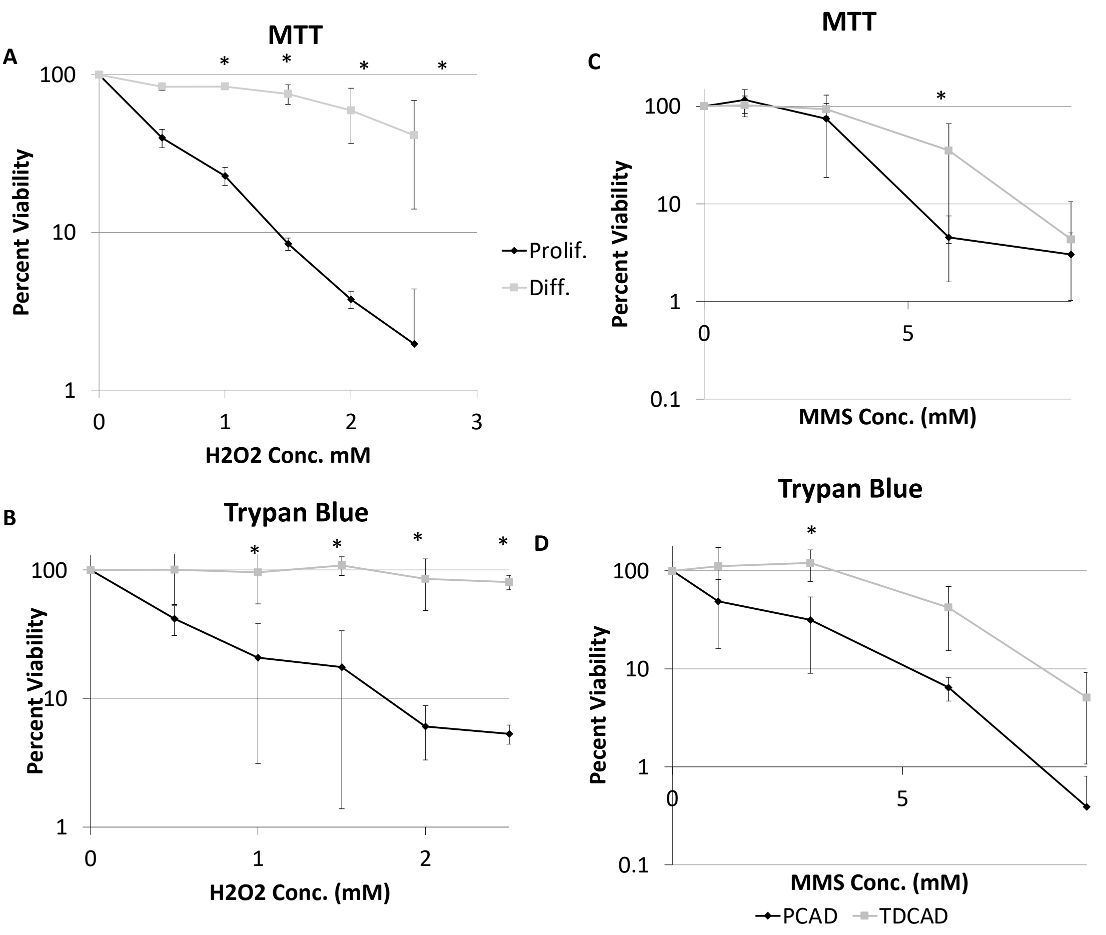

Figure S5. Viability of CAD cells challenged with H_2O_2 (**A, B**) or MMS (**C, D**). CAD cells were treated for 30 min with the indicated concentrations of H_2O_2 or MMS, transferred to fresh medium, and after 24 h assayed for viability using either the MTT reagent (**A, C**) or trypan blue dye (**B, D**). * denotes $p<0.05$ between Prolif and Diff as measured by student's t test (n=3).

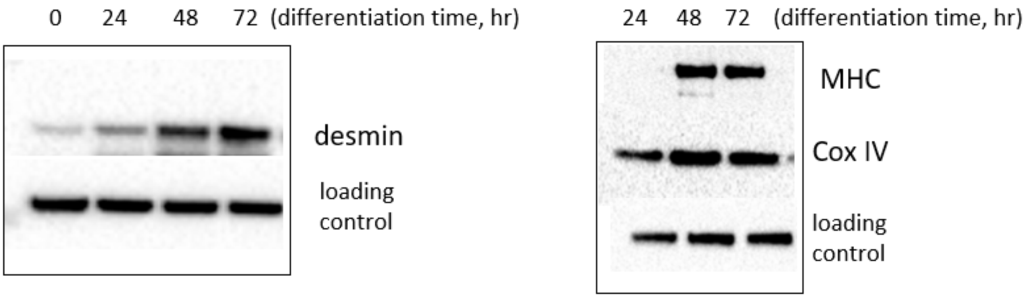

Figure S6. Western blots of mouse muscle cells. Markers of differentiation were confirmed over 72 h. Desmin is an early marker of muscle differentiation; MHC indicates that myosin heavy chain, a late marker of differentiation. The level of Cox IV was measured to show the well-known increase of mitochondria in the in the syncytia of mature muscle.
